# Rice husk ash based growing media impact on cucumber and melon growth and quality

**DOI:** 10.1038/s41598-024-55622-4

**Published:** 2024-03-01

**Authors:** Meng Li, Xian-peng Ning, Tian-tian Gao, Shazrul Fazry, Babul Airianah Othman, Ahmad Abdul Kareem Najm, Douglas Law

**Affiliations:** 1 College of Horticulture, Xinyang Agriculture and Forestry University, Xinyang, 464000 Henan China; 2 Department of Biological Sciences and Biotechnology, Faculty of Science and Technology, Universiti Kebangsaan Malaysia, 43600 Bangi, Malaysia; 3 College of Horticulture, South China Agricultural University, Guangzhou, 10564 Guangdong China; 4 Department of Food Sciences, Faculty of Science and Technology, Universiti Kebangsaan Malaysia, 43600 Bangi, Selangor Malaysia; 5 Faculty of Health and Life Sciences, INTI International University, 71800 Nilai, Negeri Sembilan Malaysia

**Keywords:** Carbohydrate metabolism, Fruit quality, Growth, Photosynthesis, Substrate formulation, Plant biotechnology, Plant development

## Abstract

Rice husk, an agricultural waste from the rice industry, can cause serious environmental pollution if not properly managed. However, rice husk ash (RHA) has been found to have many positive properties, making it a potential replacement for non-renewable peat in soilless planting. Thus, this study investigated the impact of a RHA composite substrate on the growth, photosynthetic parameters, and fruit quality of cucumber (Yuyi longxiang variety) and melon (Yutian yangjiaomi variety). The RHA, peat, vermiculite, and perlite were blended in varying proportions, with the conventional seedling substrate (peat:vermiculite:perlite = 1:1:1 volume ratio) serving as the control (CK). All plants were cultivated in barrels filled with 10L of the mixed substrates. The results from this study found that RHA 40 (RHA:peat:vermiculite:perlite = 4:4:1:1 volume ratio) significantly enhanced substrate ventilation and positively influenced the stem diameter, root activity, seedling index, chlorophyll content, net photosynthetic rate (*P*n), stomatal conductance (*G*s), and transpiration rate (*T*r) of cucumber and melon plants. Additionally, plant planted using RHA 40, the individual fruit weight of cucumber and melon found to increase by 34.62% and 21.67%, respectively, as compared to the control. Aside from that, both cucumber and melon fruits had significantly higher sucrose, total soluble sugar, vitamin C, and soluble protein levels. This subsequently improved the activity of sucrose synthase and sucrose phosphate synthase in both cucumber and melon. In conclusion, the RHA 40 found to best promote cucumber and melon plant growth, increase plant leaf photosynthesis, and improve cucumber and melon fruit quality, making it a suitable substrate formula for cucumber and melon cultivation in place of peat.

## Introduction

Rice holds significant importance in Asia, serving as a staple diet for billions of individuals. Global paddy rice production is anticipated to reach approximately 793 million tons by 2023, with China contributing 256 million tons alone^1^. Consequently, rice husk stands as a prevalent agricultural waste from the rice industry, constituting 20% of the total rice production^2^. The rice husk is usually discarded by burning it off in the field or simply dumping it in landfills or rivers, which has caused serious environmental problems^3^. The volume of rice husk waste is projected to increase concurrently with advancements in cultivation techniques, the adoption of improved machinery, and the introduction of new paddy varieties^4^.

In comparison to traditional soil cultivation, soilless cultivation methods, such as using peat, alleviate pressure on soil resources by reducing the usage of macro and micronutrients. They also conserve water, fertilizer, lower production costs, decrease labor requirements, and provide improved management of diseases and pests associated with continuous land use^5,6^. Persistent soil exploitation additionally inflicts severe damage on the local ecological environment^7^. Nonetheless, peat is costly and non-renewable. Therefore, there is a necessity to explore new cultivation mediums, such as rice husk ash (RHA), which could potentially replace or substantially reduce the reliance on peat in the industry. RHA is a repurposed ash material made from carbonized rice hulls, the utilization of rice husk in diverse applications has demonstrated positive impacts and economic value, notably in China and Malaysia, where rice is a primary crop^8^. Runkle et al.^9^ suggested that incorporating rice husks as a soil amendment can enhance the sustainability of rice production by mitigating the uptake of toxic metals and reducing water consumption. Geethakarthi et al.^10^ provided additional support, emphasizing the potential of rice husks and their ash in diverse applications such as construction, energy production, water purification, and soil stabilization. RHA exhibits loose porosity, facilitating good ventilation and water permeability. It possesses a low alkaline pH and a high potassium content, which are advantageous for plant growth^11^. Rice husk has been identified as a potential alternative to traditional substrates like peat due to its abundance and renewable nature^12^. Amor et al.^13^ discovered that dry matter and chlorophyll content increase significantly in pepper seedlings grown in substrate containing RHA. Tomato yield was found to increase significantly when cultured in a substrate mixture containing RHA^14^. These studies collectively highlight the potential of rice husk as a versatile and sustainable alternative to traditional substrates. This study aims to explore the potential of RHA as a planting substrate in soilless cultivation for Cucurbitaceae family plants, including cucumber and melon. The goal is to enhance yields, elevate dry matter and chlorophyll contents, and facilitate callus induction and rooting in the plants.

Cucumber (*Cucumis sativus* L.) and Melon (*Cucumis melo* L.), locally known as Yuyi Longxiang and Yutian Yangjiaomi, respectively, are extensively cultivated crops in China, prized for their nutrient richness and high market demand. Additionally, these crops offer major advantages, including brief cultivation periods and high input and output. Thus far, numerous studies have focused on substrate formulations for cultivating cucumber and melon seedlings, with limited research on the effects of RHA on melons, cucumbers, and other fruit crops.

This study aimed to assess the impact of a composite substrate comprising RHA on the growth, photosynthetic parameters, and fruit quality of cucumber and melon plants. The study investigated the physical and chemical properties of the blended substrates, analyzing their impact on plant growth, photosynthesis, fruit quality, and carbohydrate metabolism in cucumber and melon plants. This exploration aimed to determine the optimal substrate ratio for cultivating cucumber and melon, offering a theoretical foundation for the future development and utilization of RHA. Consequently, this research encourages rice farmers to repurpose rice husks for agricultural uses, mitigating environmental pollution. Additionally, it offers the potential to generate supplementary income for farmers typically positioned at the lower rungs of the economic ladder.

## Materials and methods

### Materials

The cucumber variety “Yuyi longxiang” and melon variety “Yutian Yangjiaomi” were purchased from Henan Yuyi Seed Co., Ltd (Henan, China). The substrates used in the experiment were RHA, peat, vermiculite, and perlite, purchased from Xinyang Shangtianti Hengyuan Mining Co., Ltd (Henan, China). The physical and chemical properties of each substrate are shown in Table 1.

**Table 1:** The physical and chemical properties of the substrate.

Substrate	Bulk density (g cm^−3^)	Total porosity (%)	Water-holding porosity (%)	Aeration porosity (%)	pH	EC (mS cm^−1^)	Organic matter (g kg^−1^)	Total N (g kg^−1^)	Total P (g kg^−1^)	Total K (g kg^−1^)
RHA	0.20	67.02	45.26	22.10	7.78	0.77	219.13	9.88	5.01	19.31
Peat	0.26	68.70	57.21	11.43	5.53	0.37	310.21	14.33	2.85	1.56
Vermiculite	0.37	72.21	57.40	13.25	6.72	0.22	3.88	0.55	0.66	4.66
Perlite	0.15	76.31	31.82	30.12	7.21	0.08	4.57	0.73	7.59	17.88

RHA, rice husk ash; EC, electrical conductivity.

### Cultivation methods

The study was conducted in the greenhouse of the Internet of Things in Xinyang Agriculture and Forestry University Intelligent Horticulture Experimental Base from July to December 2022. The experimental site is located at 114° 7′ 3″ E and 32° 9′ 51″ N, with an elevation of about 114.35 m, belonging to the transition climate from subtropical to northern temperate zone. The average day/night temperatures inside the greenhouse were 22–28 °C/16–18 °C, the light intensity was between 100 and 160 Wm^2^ (watt per square meter) and the photoperiod was between 10 and 12 h, relative humidity was maintained at 60–70% during cultivation. The conventional seedling substrate, namely peat: vermiculite: perlite (ratio 1:1:1) was used as the control CK, and RHA, peat, vermiculite, and perlite were mixed according to different proportions to form compound substrates the ratios of each RHA are shown in Table 2. Cucumber and melon seeds were germinated in a mixture of peat moss (75%) and perlite (25%) at a volume ratio of 3:1 on July 25, 2022. The seeds of both cucumber and melon were cultivated in a 50-hole substrate tray and irrigated twice daily until seedlings were produced. Subsequently, at the three-leaf stage, approximately three weeks (on August 16, 2022) after seed planting, the seedlings were transplanted into cultivation barrels (with capacity of 10 L, an upper mouth diameter of 34 cm, a lower mouth diameter of 22 cm, and a height of 28 cm) filled with mixed substrates (the plant was planted at the spacing of 30 cm, the row spacing of 50 cm and 10 seedlings was planted per square meter). Six groups of treatments were tested, and each treatment was repeated three times. Thirty plants were transplanted in each treatment, and one plant was planted in each cultivation barrel. After the cucumber and melon plants were planted and during the vegetative stage, each plant was watered with 200–500 mL of 1/2 Hoagland nutrient solution daily. During the flowering and fruiting stage, 1.0–1.5 L of Hoagland nutrient solution was applied daily for each cucumber plant, and 1.5–2.5 L of Hoagland nutrient solution applied daily for each melon plant. The pH and electrical conductivity (EC) of the nutrient solution were monitored daily to ensure an average pH value between 6.2 and 6.4, regulated with Hydrochloric acid (HCL, Huabo Ltd, China). Furthermore, the EC was maintained within the range of 1500–3000 μS microsiemens.

**Table 2:** Different RHA substrate formulations (volume ratio).

Treatment	RHA	Peat	Vermiculite	Perlite
CK	0	33.3	33.3	33.3
RHA 20	20	60	10	10
RHA 30	30	50	10	10
RHA 40	40	40	10	10
RHA 50	50	30	10	10
RHA 60	60	20	10	10

### Substrate physicochemical properties measurement

The physical and chemical properties of the substrate, such as bulk density, porosity, and water-holding pore, were determined according to the method of Li et al.^15^. EC and pH were measured by method described by Adebajo et al.^14^.

### Microbial count measurement

After cultivation, the substrate samples were taken to determine the microorganism population. The dilution plate method^6^ was used for detection and counting. Bacteria suspension with a dilution of 10^–4^, 10^−5^ and 10^–6^ was cultured in beef paste peptone medium. Actinomycetes and fungi were cultured in modified Gao's No.1 culture medium and PDA with 10^–3^, 10^–4^, and 10^–5^ dilutions.

### Plant growth measurement

Three plants were selected randomly from each replication for all treatments. Plant height and root length were measured with a tape meter. The stem diameter was measured with a vernier caliper (Mitutoyo CD-15APX, Japan). The leaf area, root volume, root activity of seedlings and seedling index were measured by method described by Li et al.^15^. Plant fresh and dry weight were also measured at the end of experiments by analytical balance precision 0.001 (Isvart CXE-1, USA). The plant of materials dried in oven (Nabertherm TR 1050, Germany) at 60 °C for 72 h.

### Chlorophyll content measurement

Chlorophyll was analyzed using the ethanol and acetone mixture method^16^. Briefly, at the full flowering stage of the plant, the first fully expanded functional leaf under the growth point of each treatment was selected, cucumber and melon leaves were first cut into pieces, after which a vein was removed. Samples were then weighed, and a 0.1 g sample was put into a 25 mL volumetric flask, mixed with 10 mL of mixed extractive solution, and stored in the dark. After the leaves were turned white, samples were mixed with an extraction reagent to a constant volume of 25 mL. The extraction reagent was blank, and the spectrophotometer (Diones 4X250, USA) was used for colorimetric determination at the wavelengths of 663, 646, and 470 nm, respectively, each treatment was repeated three times. The leaf color index was calculated using the following formula: total chlorophyll/carotenoid^15^.

### Photosynthesis rate measurement

In the full flowering period of cucumber and melon, the first fully expanded functional leaf under the growth point of each treatment was selected, and the photosynthetic gas exchange parameters, including net photosynthetic rate* (P*n*)*, stomatal conductance *(G*s*)*, intercellular CO_2_ concentration *(C*i*)* and transpiration rate *(T*r*)*, were measured by portable photosynthesis measurement System (Li-6400, USA) at 9:00–11:00 am^17^, three plants were determined for each treatment.

### Fruit quality and yield measurement

The solid soluble of fruit was measured by LH-B55 Saccharimeter (Lu Heng Biological Co. Ltd, China). Sucrose, total soluble sugar, and starch were determined according to the method described by Zhai et al.^18^, vitamin C was determined by 2,6-dichloroindophenol titration^19^, soluble protein was determined by Coomassie brilliant blue G-250 staining^20^, nitrate was determined by method described by Li et al^15^. Vernier caliper (Mitutoyo CD-15APX, Japan) was used to measure the transverse and longitudinal diameter of fruit, fruit shape index was calculated using the following formula: longitudinal diameter/transverse diameter, the weight per fruit and yield per plant were measured by electronic balance (Aikl Instruments Ltd, WT3002LB, China).

### Fruit carbohydrate metabolism measurement

Sucrose synthase (SS) activity and sucrose phosphate synthase (SPS) activity were measured according to the method of Wang et al.^21^, the activity of soluble acid invertase (AI) was determined according to the method of Song et al.^22^.

### Data analysis

The results were expressed as the means value ± standard error and were calculated and statistically examined using an analysis of variance (ANOVA) and a Duncan’s multiple range test. Statistical significance was considered at *P* < 0.05, unless otherwise stated. Most tests were conducted using SPSS version 23.0 (IBM SPSS, Armonk, NY: IBM Corp.). The data underwent rigorous testing for statistical significance.

### Ethics approval

All authors declared that all methods and protocols were carried out by relevant guidelines and regulations.

## Results

### Physical and chemical properties of the substrate before cultivation

The bulk density of the substrate in each treatment ranged between 0.17 and 0.23 g cm^−3^; the bulk density of the substrate in RHA 20 was larger compared to other substrates (*P* < 0.05) (Table 3). With the increase of RHA content and the decrease of peat content, the total porosity of the substrate in each treatment showed an upward trend, while the water-holding pores showed a downward trend (Table 3). The total porosity and air gap of RHA 50 showed the highest increase compared to other treatments (it increased by 18.88% and 98.32%, respectively, compared to CK) (Table 3).

**Table 3:** Physical and chemical properties of the substrate before cultivation.

Treatment	Bulk density (g cm^−3^)	Total porosity (%)	Water-holding porosity (%)	Aeration porosity (%)	pH	EC (mS cm^−1^)
CK	0.21 ± 0.01 c	54.97 ± 0.03 c	36.49 ± 0.07 a	15.48 ± 0.07 f	6.74 ± 0.02 b	1.49 ± 0.34 c
RHA 20	0.23 ± 0.01 a	56.96 ± 0.12 d	35.67 ± 0.03 b	17.37 ± 0.02 e	6.35 ± 0.01 d	1.62 ± 0.32 bc
RHA 30	0.22 ± 0.01 b	61.21 ± 0.37 c	34.82 ± 0.02 c	27.48 ± 0.01 d	6.45 ± 0.01 c	1.87 ± 0.22 bc
RHA 40	0.19 ± 0.02 d	64.07 ± 0.02 b	34.64 ± 0.15 c	29.50 ± 0.01 c	6.85 ± 0.02 b	2.01 ± 0.33 b
RHA 50	0.18 ± 0.03 e	65.35 ± 0.20 a	34.59 ± 0.12 c	30.70 ± 0.18 a	7.02 ± 0.02 b	2.08 ± 0.27 ab
RHA 60	0.17 ± 0.02 f	65.28 ± 0.02 a	33.74 ± 0.37 d	29.61 ± 0.02 b	7.12 ± 0.03 a	2.34 ± 0.31 a

The mean value ± standard error in the same column with dissimilar letters are significantly different at *P* < 0.05.

The results also showed that the substrate's aeration was improved after adding RHA. The pH value of each treatment ranged between 6.0 and 7.0, thus meeting the requirements of a soilless culture medium. With the increase of RHA content, the EC value of each treatment showed an upward trend, where the EC value of the RHA 60 was the largest (Table 3).

### Physical and chemical properties of the substrate after cultivation

The substrate bulk density of cultivated cucumber was 0.18–0.25 g cm^−3^, and that of melon was 0.19–0.27 g cm^−3^ (Table 4). The bulk density of cucumber and melon treated with RHA 20 was the highest vs. other treatments (*P* < 0.05). The water-holding void, aeration void, and total porosity of cucumber substrate under RHA 40 showed the highest increase (25.21%, 3.25%, and 15.58%, respectively, compared with CK) (Table 4).

**Table 4:** Physical and chemical properties of the substrate after cultivation.

Plant	Treatment	Bulk density (g cm^−3^)	Total porosity (%)	Water-holding porosity (%)	Aeration porosity (%)	pH	EC (mS cm^−1^)
Cucumber	CK	0.18 ± 0.03 e	58.21 ± 0.03 e	32.68 ± 0.01 f	25.53 ± 0.01 c	6.25 ± 0.06 c	1.65 ± 0.21 ab
	RHA 20	0.25 ± 0.02 b	58.13 ± 0.02 f	40.74 ± 0.03 c	17.39 ± 0.01 f	6.13 ± 0.01 d	1.68 ± 0.26 ab
	RHA 30	0.25 ± 0.01 b	61.94 ± 0.04 c	42.97 ± 0.01 a	18.97 ± 0.03 e	6.21 ± 0.01 c	0.97 ± 0.17 c
	RHA 40	0.24 ± 0.03 a	67.28 ± 0.03 a	40.92 ± 0.02 b	26.36 ± 0.01 a	6.31 ± 0.01 b	0.98 ± 0.18 c
	RHA 50	0.20 ± 0.01 c	64.14 ± 0.04 b	38.02 ± 0.02 e	26.12 ± 0.02 b	6.23 ± 0.01 c	1.72 ± 0.24 a
	RHA 60	0.19 ± 0.02 d	61.54 ± 0.02 d	38.58 ± 0.03 d	22.96 ± 0.03 d	6.81 ± 0.01 a	1.21 ± 0.19 b
Melon	CK	0.26 ± 0.01 b	66.01 ± 0.01 b	51.37 ± 0.03 c	15.12 ± 0.05 d	6.10 ± 0.02 d	0.32 ± 0.02 d
	RHA 20	0.27 ± 0.03 a	65.73 ± 0.03 b	51.04 ± 0.02 c	16.67 ± 0.05 c	6.17 ± 0.02 c	1.52 ± 0.03 c
	RHA 30	0.25 ± 0.01 b	67.16 ± 1.00 a	52.69 ± 0.97 b	16.48 ± 0.13 c	6.20 ± 0.02 b	1.44 ± 0.01 c
	RHA 40	0.25 ± 0.13 a	67.69 ± 0.17 a	53.58 ± 0.15 a	16.32 ± 0.05 c	6.21 ± 0.02 b	1.25 ± 0.01 a
	RHA 50	0.19 ± 0.05 c	63.10 ± 0.01 d	42.40 ± 0.04 e	21.70 ± 0.05 a	6.24 ± 0.01 a	1.14 ± 0.03 b
	RHA 60	0.19 ± 0.02 c	64.40 ± 0.04 c	46.77 ± 0.01 d	17.63 ± 0.03 b	6.26 ± 0.02 a	0.35 ± 0.11 b

The mean value ± standard error in the same column with dissimilar letters are significantly different at *P* < 0.05.

The total porosity and water-holding porosity of the RHA 40 showed the highest increase (2.55% and 4.3%, respectively) compared with the control (Table 4). With the increase in RHA content, the pH of cucumber and melon substrates showed an increasing trend (Table 4). The conductivity of cucumber reached the maximum in RHA 50 (increased by 4.24% compared with CK), and the conductivity of melon reached the maximum of 1.52 mS cm^−1^ in RHA 20. Generally speaking, compared with before cultivation, the bulk density of the substrate generally increased, which may due to the process of spraying a nutrient solution or collecting and air-drying substrate during cultivation; However, the EC value generally decreased, which may be due to the absorption of nutrient elements and water leaching by crops.

### The number of microorganisms in the substrate

The number of microorganisms in the CK group without RHA was the highest vs. other treatments (Table 5). The number of fungi and actinomycetes in cucumber and melon treated with CK was about 1–3 times that of other treatments. With the increase of RHA content, the number of microorganisms in all treated substrates decreased, and the number of microorganisms in RHA 60 was the lowest. Compared with CK treatment, the number of actinomycetes, bacteria, fungi, and microorganisms in cucumber treated by RHA 60 decreased by 48.91%, 49.58%, 69.10%, and 52.47%, respectively (Table 5). Moreover, the number of microorganisms treated with substrate RHA 60 was the lowest, which decreased by 48.44%, 56.67%, 70.14%, and 56.95%, respectively, compared to CK treatment (all* P* < 0.05). To sum up, after adding a proper amount of RHA to the substrate, the number of actinomycetes, bacteria, fungi, and microorganisms decreased compared with CK.

**Table 5:** Number of microorganisms in substrate.

Plant	Treatment	Quantity of actinomycetes (*10^4^ CFU g^−1^)	Number of bacteria (*10^4^ CFU g^−1^)	Fungi quantity (*10^4^ CFU g^−1^)	Total microbial quantity (*10^4^ CFU g^−1^)
Cucumber	CK	3.66 ± 0.09 a	7.25 ± 0.31 a	2.88 ± 0.16 a	13.55 ± 0.23 a
	RHA 20	2.79 ± 0.23 b	5.32 ± 0.23 b	1.78 ± 0.10 b	9.89 ± 0.18 b
	RHA 30	2.57 ± 0.18b c	5.03 ± 0.32 b	1.52 ± 0.14 b	9.12 ± 0.36 b
	RHA 40	2.48 ± 0.12 bc	4.68 ± 0.45 bc	1.34 ± 0.09 bc	8.50 ± 0.21 c
	RHA 50	1.92 ± 0.06 c	4.37 ± 0.33 c	1.21 ± 0.16 b	7.36 ± 0.25 c
	RHA 60	1.87 ± 0.11 d	3.68 ± 0.27 c	0.89 ± 0.02 c	6.44 ± 0.16 d
Melon	CK	3.20 ± 0.10 a	6.67 ± 0.05 a	2.21 ± 0.06 a	12.08 ± 0.22 a
	RHA 20	2.36 ± 0.15 b	4.56 ± 0.10 ab	1.53 ± 0.05 b	8.45 ± 0.20 b
	RHA 30	2.27 ± 0.20 b	4.23 ± 0.25 b	1.31 ± 0.05 b	7.81 ± 0.45 bc
	RHA 40	2.18 ± 0.35 bc	3.77 ± 0.70 b	1.27 ± 0.03 c	7.22 ± 0.26 c
	RHA 50	1.82 ± 0.25 c	3.23 ± 0.40 c	1.02 ± 0.15 cd	6.07 ± 0.37 c
	RHA 60	1.65 ± 0.10 c	2.89 ± 0.55 e	0.66 ± 0.20 d	5.20 ± 0.16 d

The mean value ± standard error in the same column with dissimilar letters are significantly different at *P* < 0.05.

### Effects of different substrate ratios on the growth of cucumber and melon plants

Plant height is an important index used to measure the growth speed of plants, and stem diameter can reflect the robustness of seedlings to a certain extent. Different contents of RHA have different effects on the growth of cucumber plants (Table 6). The plant height and stem diameter of cucumber plants were the highest after RHA 40, increasing by 5.57% and 10.69%, respectively, compared with CK. Moreover, the plant height and stem diameter of melon treated with RHA 40 were significantly higher than those of CK, increasing by 13.73% and 8.43%, respectively (Table 6).

**Table 6:** Effects of different substrate ratios on the growth of cucumber and melon plants.

Plant	Treatment	Plant height (cm)	Stem diameter (mm)	Root length (mm)	Root volume (cm^3^)	Leaf area (cm^2^)	Fresh weight (g)	Dry weight (g)
Cucumber	CK	163.25 ± 3.17 c	12.35 ± 0.32 c	52.64 ± 3.82 d	15.38 ± 0.27 c	221.31 ± 11.05 d	363.57 ± 25.31 c	35.47 ± 1.76 c
	RHA 20	166.73 ± 3.45 b	12.34 ± 0.34 bc	55.16 ± 6.12 c	16.12 ± 2.13 b	230.63 ± 8.99 c	413.46 ± 19.56 a	40.32 ± 2.09 c
	RHA 30	162.27 ± 2.56 c	13.14 ± 0.45 a	57.28 ± 4.40 bc	15.34 ± 0.56 c	235.67 ± 10.35 c	384.30 ± 21.09 b	39.45 ± 1.77 b
	RHA 40	172.34 ± 3.67 a	13.67 ± 0.16 a	58.62 ± 4.27 b	17.58 ± 0.17 a	268.19 ± 11.25 a	380.32 ± 20.65 b	43.47 ± 2.78 a
	RHA 50	167.53 ± 5.78 b	12.59 ± 0.23 b	66.06 ± 3.18 a	17.10 ± 1.45 ab	263.57 ± 9.88 ab	373.65 ± 24.01b c	37.10 ± 2.18 bc
	RHA 60	160.17 ± 4.12 d	11.64 ± 0.10 c	51.76 ± 5.02 d	15.27 ± 1.09 c	228.79 ± 10.03 cd	361.14 ± 26.45 d	32.16 ± 2.98 d
Melon	CK	147.23 ± 2.01 d	9.02 ± 0.29 bc	98.78 ± 7.35 c	10.46 ± 0.38 c	213.01 ± 10.76 cd	351.82 ± 20.34cd	34.38 ± 1.87 c
	RHA 20	163.20 ± 5.12 b	9.21 ± 0.17 bc	102.23 ± 6.44 bc	11.12 ± 1.01 b	234.32 ± 10.67 b	359.98 ± 20.10bc	39.56 ± 2.01 b
	RHA 30	160.10 ± 4.23 b	9.33 ± 0.33 b	110.88 ± 6.54 a	10.51 ± 0.28 bc	231.45 ± 12.87 b	369.82 ± 25.20b	40.60 ± 2.54 b
	RHA 40	167.45 ± 2.56 a	9.78 ± 0.43 a	107.56 ± 5.52 b	12.67 ± 0.35 a	263.24 ± 12.78 a	367.10 ± 21.54b	42.17 ± 2.00 a
	RHA 50	161.15 ± 3.12 b	9.56 ± 0.28 a	106.34 ± 8.12 b	12.34 ± 0.34 ab	256.23 ± 11.23 ab	404.13 ± 27.63a	35.79 ± 2.16 c
	RHA 60	154.77 ± 2.82 c	8.12 ± 0.17 c	95.12 ± 7.26 c	10.23 ± 0.54 d	225.21 ± 10.23 c	351.15 ± 30.12cd	31.50 ± 2.31 d

The mean value ± standard error in the same column with dissimilar letters are significantly different at *P* < 0.05.

The root length of cucumber plants under different treatments ranged from 51.76 to 66.06 cm, with the RHA 50 having the largest value (66.06 cm). On the other hand, the root length of melon under RHA 30 was the longest, reaching 110.88 cm (12.25% higher than that of the CK) (Table 6).

The root volume and leaf area of cucumber plants treated with RHA 40 were the largest; compared with the control, these values increased by 14.30% and 21.19%, respectively. The root volume and leaf area of melon in RHA 40 were the largest, which were 12.67 cm^3^ and 263.24 cm^3^, which increased by 21.13% and 23.58% compared with the CK (Table 6).

The fresh weight of cucumber per plant was the highest in RHA 20, which was 413.46 g. The fresh weight of melon per plant was the highest in the RHA 50, which was 404.13 g. The dry weight of the cucumber plant in RHA 40 was the largest. With the increase of RHA content, the dry weight of melon per plant increased at first and then decreased, reaching the peak value of 42.17 g in the RHA 40 (Table 6).

To sum up, adding a proper amount of RHA is beneficial to promoting the growth and dry matter accumulation of cucumber and melon plants, and the effect is more obvious when the RHA is added at 40%.

### Effects of different substrate ratios on root activity and seedling index of cucumber and melon plants

**Figure 1:**
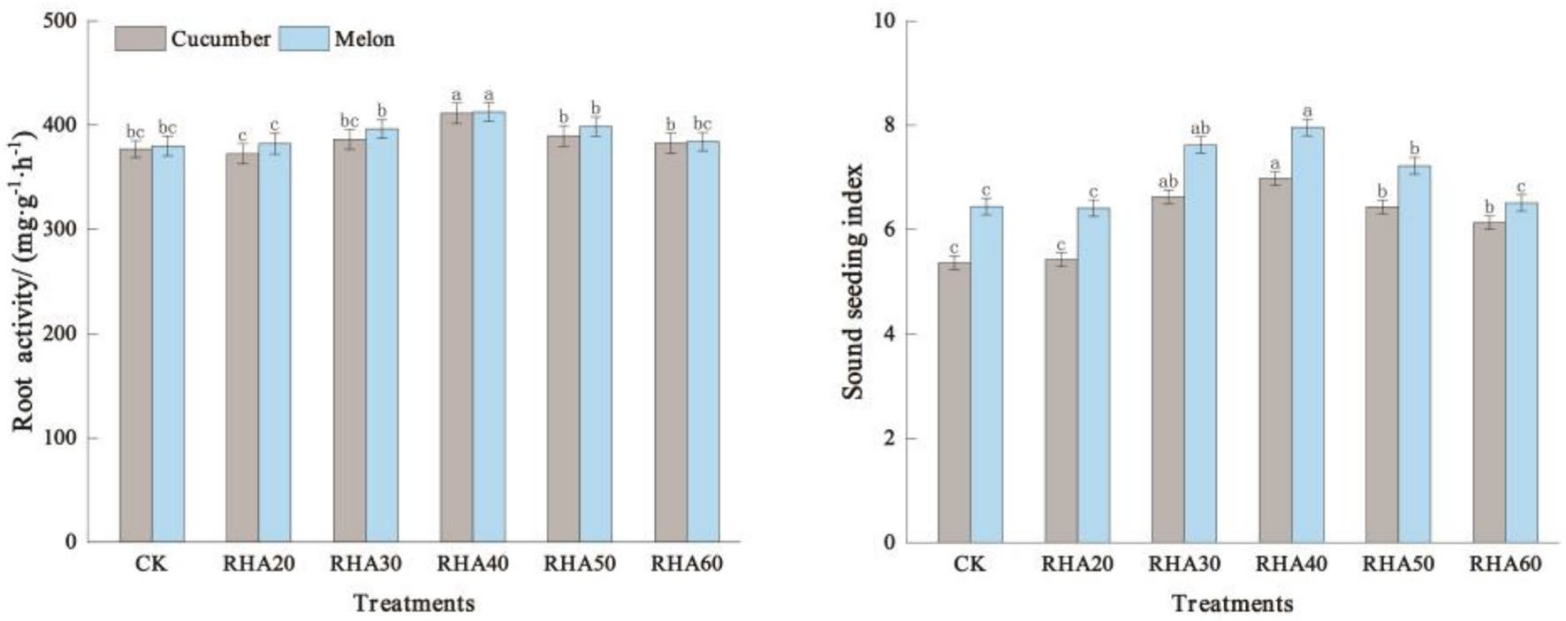
Effects of different substrate ratios on root activity and seedling index of cucumber and melon. Error bars represent the standard deviation calculated from 3 replicates. Bars followed by the same letter are not significantly different at *P* < 0.05.

The effects of different treatments on the root vigor of cucumber showed varying degrees with the increase of the hull ash content. The RHA 40 reached the maximum, i.e., 411.36 mg g^−1^ h^−1^ with an increase of 9.12% compared with CK. There was no significant difference between treatments RHA 20, RHA 60, and CK. The seedling strength index reached a maximum of 6.98 in RHA 40, and the seedling strength index was RHA 40 > RHA 30 > RHA 50 > RHA 60 > RHA 20 > CK; RHA 50, RHA 60, RHA 20, RHA 30, and RHA 40 were increased by 30.22%, 23.69%, 19.96%, 14.37%, and 1.12% compared with CK, respectively (Fig. 1).

For melon, the root vigor of RHA 40 was the highest, which was 8.61% higher than that of CK (Fig. 1). Also, adding a certain amount of RHA can improve the root vigor and seedling strength index of cucumber and melon to varying degrees; the best effect was seen when the content of RHA was 40%.

### Effects of different substrate ratios on photosynthetic pigment content in cucumber and melon leaves

**Figure 2:**
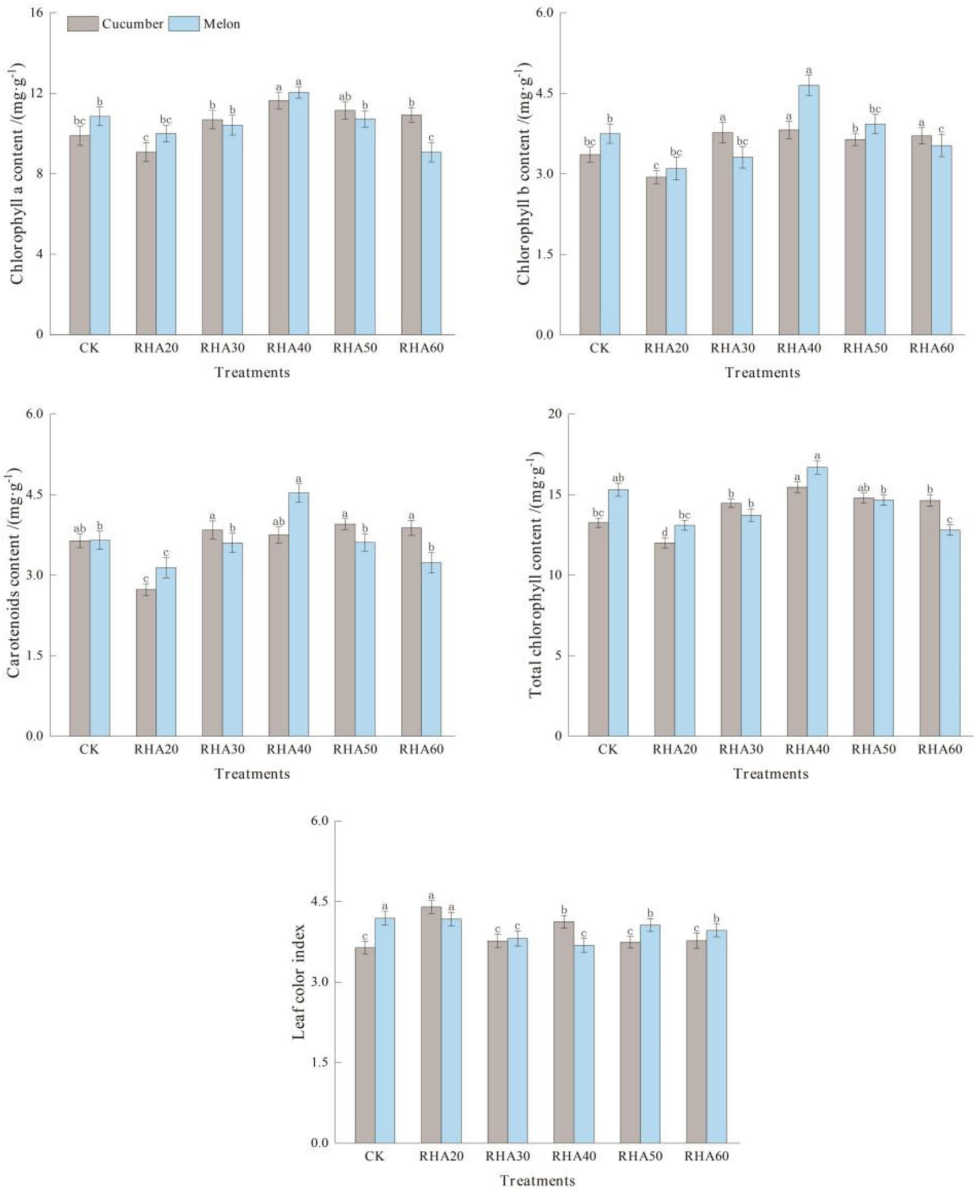
Effects of different substrate ratios on photosynthetic pigment content in cucumber and melon leaves. Error bars represent the standard deviation calculated from 3 replicates. Bars followed by the same letter are not significantly different at *P* < 0.05.

With the increase of RHA content for cucumber, the chlorophyll in RHA 40 reached the maximum value of 11.64 mg g^−1^, which increased by 17.69% compared with CK (Fig. 2). The chlorophyll a content in RHA 20 reached the minimum value of 9.07 mg g^−1^, which decreased by 8.29%% compared with CK. Compared with CK, the chlorophyll b content of RHA 40 increased by 13.69%. The total chlorophyll content reached the maximum in RHA 40, which increased by 16.68% compared with CK (Fig. 2). However, carotenoids reached the peak value in RHA 50, which increased by 8.52% compared with CK. The maximum leaf color index of RHA 20 was 4.40, and the minimum of CK was 3.64.

For melon, chlorophyll a, chlorophyll b, total chlorophyll, and carotenoids reached the maximum in RHA 40, which was 12.04 mg g^−1^, 4.65 mg g^−1^, 16.69 mg g^−1^ and 4.53 mg g^−1^, respectively, increasing by 10.97%, 24%, 9.08%, and 24.11% compared with CK (all *P* < 0.05) (Fig. 2). The maximum leaf color index of RHA 20 was 4.17, which was significantly different from that of treatments RHA 30, RHA 40, RHA 50, and RHA 60 (all *P* < 0.05) (Fig. 2).

These data suggested that when the content of RHA was 40%, the chlorophyll content of cucumber and melon was the highest.

### Effects of different substrate ratios on photosynthetic parameters of cucumber and melon leaves

**Figure 3:**
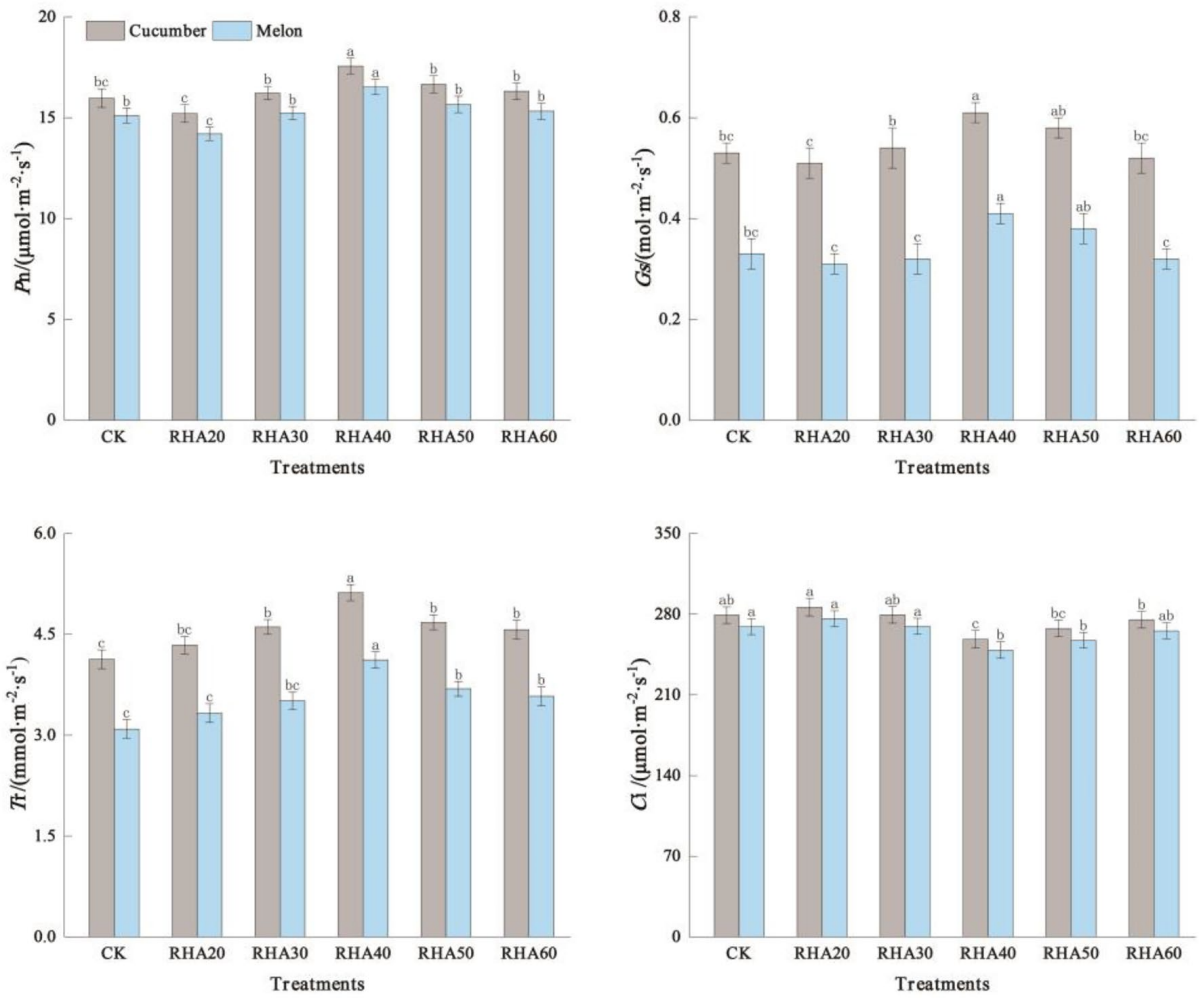
Effects of different substrate ratios on photosynthetic parameters of cucumber and melon leaves. Error bars represent the standard deviation calculated from 3 replicates. Bars followed by the same letter are not significantly different at *P* < 0.05.

The net photosynthetic rate (*P*n), stomatal conductance (*G*s), and transpiration rate (*T*r) of cucumber showed an increasing trend with the increase of the RHA content; they reached the maximum in the RHA 40 (17.56 μmol m^−2^ s^−1^, 0.16 μmol m^−2^ s^−1^ and 5.12 μmol m^−2^ s^−1^ respectively, i.e., increasing 9.96%, 15.09%, and 23.97% compared with CK; all *P* < 0.05) (Fig. 3). Contrary, the net photosynthetic rate (*P*n) and stomatal conductance (*G*s) in RHA 20 were the lowest (15.21 μmol m^−2^ s^−1^ and 0.51 μmol m^−2^ s^−1^, respectively, but showed no significant difference (*P* > 0.05) compared to CK) (Fig. 3). The intercellular CO_2_ concentration (*C*i) was the highest in RHA 20, which was 286.12 μmol m^−2^ s^−1^, and the lowest in RHA 40 was 258.29 μmol m^−2^ s^−1^(Fig. 3).

For melon, the net photosynthetic rate (*P*n), stomatal conductance (*G*s), and transpiration rate (*T*r) of RHA 40 were the largest (16.52 μmol m^−2^ s^−1^, 0.41 μmol m^−2^ s^−1^, and 4.12 μmol m^−2^ s^−1^, respectively, increasing 9.33%, 24.24%, and 33.33% compared with CK; all *P* < 0.05) (Fig. 3). The intercellular CO_2_ concentration (*C*i) reached the maximum in RHA 20 (276.13 μmol m^−2^ s^−1^) and the minimum in RHA 40 (248.92 μmol m^−2^ s^−1^) (Fig. 3). The results showed that the net photosynthetic rate (*P*n), stomatal conductance (*G*s), and transpiration rate (*T*r) of cucumber and melon were the highest when the RHA content was 40%, and the photosynthetic capacity was the strongest.

### Effects of different substrate ratios on fruit quality of cucumber and melon

There were some differences in sucrose and soluble total sugar contents of cucumber and melon among different substrates. The content of sucrose and total soluble sugar in cucumber fruit treated with RHA 40 was the highest (*P* < 0.05 vs. other treatments), followed by that treated with RHA 50. The contents of sucrose and total soluble sugar of melon in RHA 40 were 6.77 mg g^−1^ and 119.47 mg g^−1^, which were significantly higher than those in CK (increased by 11.35% and 24.93%, respectively vs CK group) (Table 7).

**Table 7:** Effects of different substrate ratios on fruit quality of cucumber and melon.

Plant	Treatment	Sucrose (mg g^−1^)	Total soluble sugar (mg g^−1^)	Starch (mg g^−1^)	Vitamin C (mg g^−1^)	Soluble protein (mg g^−1^)	Soluble solid (%)	Nitrate (mg g^−1^)
Cucumber	CK	1.66 ± 0.14 b	24.02 ± 0.71 b	6.25 ± 0.30 a	0.13 ± 0.04 bc	2.18 ± 0.20 bc	12.10 ± 0.37 ab	1.07 ± 0.05 b
	RHA 20	1.53 ± 0.22 c	22.23 ± 0.23 c	6.40 ± 0.23 a	0.14 ± 0.03 bc	2.20 ± 0.15 bc	12.01 ± 0.18 ab	1.14 ± 0.06 ab
	RHA 30	1.72 ± 0.11 b	26.65 ± 0.56 b	6.34 ± 0.15 b	0.17 ± 0.01 b	2.24 ± 0.27 b	11.04 ± 0.27 b	1.15 ± 0.03 ab
	RHA 40	2.14 ± 0.21 a	32.45 ± 0.16 a	5.65 ± 0.16 c	0.22 ± 0.04 a	3.14 ± 0.17 a	11.23 ± 0.41 b	1.01 ± 0.01 c
	RHA 50	2.03 ± 0.15 a	29.12 ± 0.28 a	6.17 ± 0.21 bc	0.18 ± 0.02 b	2.36 ± 0.03 b	12.56 ± 0.36 a	1.17 ± 0.03 a
	RHA 60	1.54 ± 0.16 c	23.71 ± 0.60 bc	6.47 ± 0.20 a	0.16 ± 0.02 b	2.21 ± 0.13 b	11.15 ± 0.46 b	1.09 ± 0.07 b
Melon	CK	6.08 ± 0.22 c	95.63 ± 0.23 d	4.20 ± 0.20 a	0.18 ± 0.01 b	6.20 ± 0.25 c	14.34 ± 0.26 ab	1.18 ± 0.03 a
	RHA 20	6.31 ± 0.31 bc	108.30 ± 0.26 bc	4.23 ± 0.11 a	0.19 ± 0.01 b	6.53 ± 0.21 b	13.21 ± 0.21 bc	1.13 ± 0.3 ab
	RHA 30	6.47 ± 0.20 b	116.12 ± 0.18 b	4.01 ± 0.25 b	0.20 ± 0.04 ab	6.34 ± 0.17 b	13.30 ± 0.19 b	1.15 ± 0.01 a
	RHA 40	6.77 ± 0.17 a	119.47 ± 0.33 a	3.18 ± 0.17 d	0.22 ± 0.02 a	7.77 ± 0.30 a	13.45 ± 0.27 b	1.03 ± 0.02 c
	RHA 50	6.32 ± 0.25 bc	106.01 ± 0.27 bc	3.34 ± 0.12 c	0.22 ± 0.07 a	7.01 ± 0.33 ab	14.51 ± 0.25 a	1.10 ± 0.03 b
	RHA 60	5.87 ± 0.19 d	101.26 ± 0.37 c	4.12 ± 0.13 ab	0.20 ± 0.08 ab	6.16 ± 0.20 c	14.22 ± 0.30 b	1.13 ± 0.01 ab

The mean value ± standard error in the same column with dissimilar letters are significantly different at *P* < 0.05.

With the increase of RHA content, the starch content of cucumber first decreased and then increased (Table 7). The starch content of cucumber treated with E was the highest, followed by RHA 20; yet, there was no significant difference between RHA 40 and CK, and the starch content of cucumber treated with C was the lowest with a decrease of 9.60% compared with CK. RHA 20 had the highest starch content in melon, while RHA 40 had the lowest content (i.e., 3.18 mg g^−1^). The starch content decreased by 22.28% compared with CK (*P* < *0.05*).

With the increase of RHA content, the vitamin C content and soluble protein content of each treatment increased to different degrees compared with the control (Table 7). The vitamin C and soluble protein content of cucumber and melon treated with C were the highest while the nitrate content was the lowest, which decreased by 5.60% and 12.71% compared with CK. The changing trend of the solid soluble content of cucumber and melon was basically the same as for the cucumber; the soluble solids content of RHA 40 was the highest, and there was no significant difference between C RHA 20nd CK treatment (Table 7).

To sum up, adding a proper amount of RHA to the substrate can improve the fruit quality and nutritional value of cucumber and melon; the RHA 40 showed the best effect.

### Effects of different substrate ratios on key enzyme activities of carbohydrate metabolism in cucumber and melon fruits

**Figure 4:**
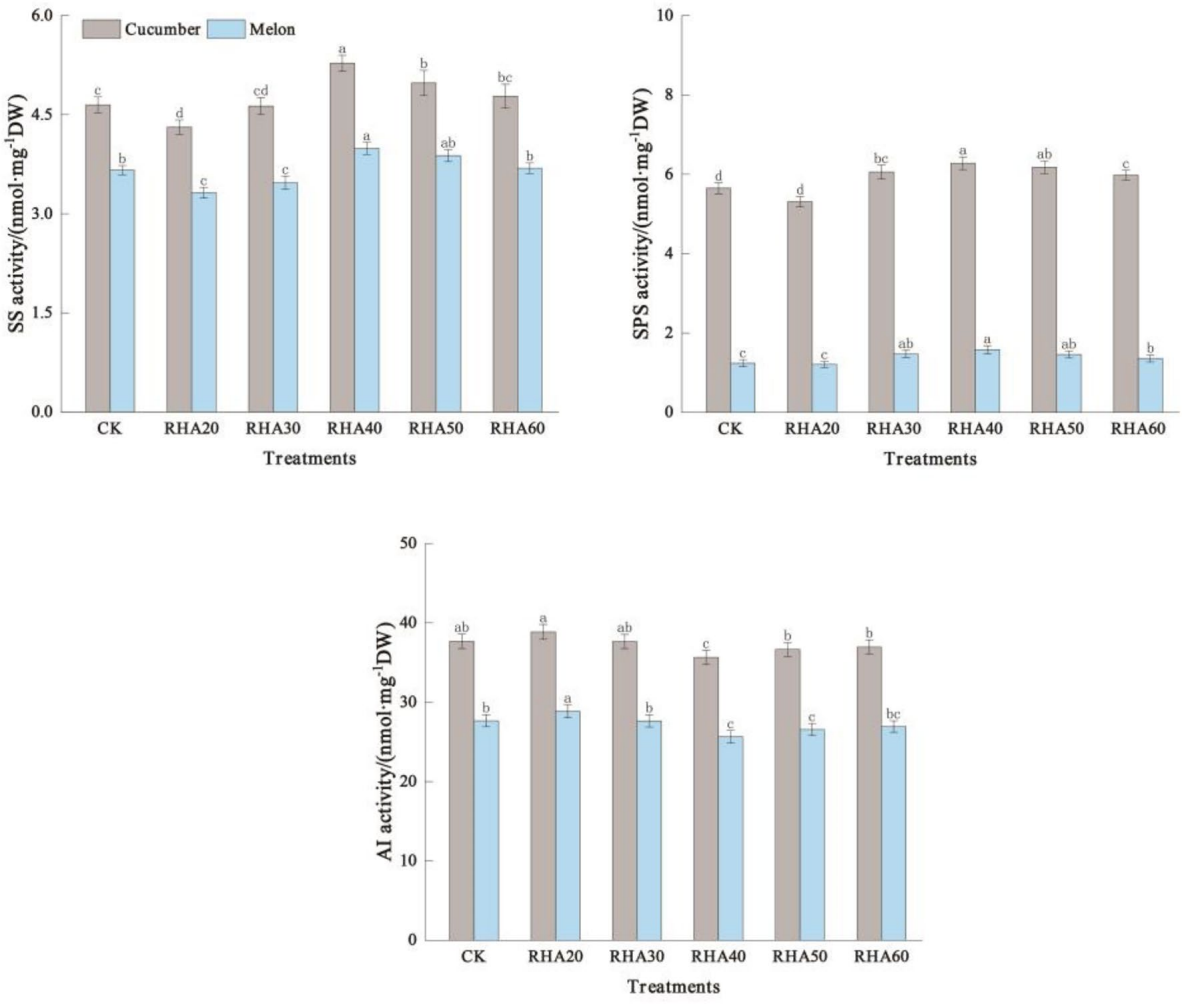
Effects of different substrate ratios on key enzyme activities of carbohydrate metabolism in cucumber and melon fruits. Error bars represent the standard deviation calculated from 3 replicates. Bars followed by the same letter are not significantly different at *P* < 0.05.

With the increase of RHA content, the sucrose synthase activity and sucrose phosphate synthase activity of cucumber reached the maximum in RHA 40, which were 5.28 nmol mg^−1^ dw and 6.28 nmol mg^−1^ dw, respectively (i.e., 13.55% and 11.15% compared with CK treatment; *P* < 0.05) (Fig. 4). The acid invertase activity of RHA 20 was the highest at 38.86 nmol mg^−1^ DW, and the lowest in RHA 40 was 35.65 nmol mg^−1^ DW (Fig. 4).

For melon, the changing trend of sucrose synthase activity and sucrose phosphate synthase activity with the increase of hull ash content was basically the same as that of cucumber and reached the maximum in RHA 40, which increased by 9.02% and 27.42%, respectively, compared with CK (*P* < 0.05) (Fig. 4). The acid invertase activity in the RHA 40 was the lowest, which was 25.64 nmol  mg^−1^ DW (7.30% lower than CK) (Fig. 4).

To sum up, the content of RHA was 40%, and the activities of sucrose synthase and sucrose phosphate synthase were the highest in cucumber and melon, which was beneficial to the accumulation of carbohydrates in fruits.

### Effects of different substrate ratio on fruit yield of cucumber and melon

There were some differences in the effects of different substrate ratios on the fruit yield of cucumber and melon. The transverse and longitudinal diameters of the cucumber and melon fruits were the largest in the RHA 40, which was significantly higher than that of the control CK (an increase of 15.03% and 11.37%; 20.22% and 21.13%, respectively, vs. CK treatment) (Table 8). There was no significant difference in transverse and longitudinal diameters of cucumber and melon when RHA 40 was applied. Also, there was no significant difference in fruit shape index among different substrates (Table 8). The single fruit weight of cucumber and melon was the highest in RHA 40, which had no significant difference with RHA 50, and they were all significantly higher than the CK treatment (*P* < 0.05). With the increase of RHA content, the yield per plant of each treatment increased to different degrees compared with the control (Table 8). The yield per cucumber plant was the highest in RHA 40, followed by RHA 50, which increased by 38.20% and 23.60% compared with CK. The yield per plant of melon was the highest in RHA 40, which had no significant difference with RHA 30 and RHA 50, and increased by 17.92% and 16.6% compared with CK (Table 8).

**Table 8:** Effects of different substrate ratios on fruit yield of cucumber and melon.

Plant	Treatment	Transverse diameter (cm)	Longitudinal diameter (cm)	Fruit shape index	Fruit weight/(kg)	Yield per plant (kg)
Cucumber	CK	3.66 ± 0.10 bc	27.45 ± 0.19 b	7.50 ± 0.22 a	0.26 ± 0.09 c	0.89 ± 0.20 b
	RHA 20	3.82 ± 0.12 b	28.17 ± 0.24 b	7.37 ± 0.21 ab	0.28 ± 0.01 b	0.97 ± 0.03 b
	RHA 30	3.69 ± 0.23 b	27.31 ± 0.32 b	7.40 ± 0.19 a	0.31 ± 0.04 a	1.08 ± 0.02 b
	RHA 40	4.31 ± 0.17 a	30.57 ± 0.37 a	7.26 ± 0.20 ab	0.35 ± 0.02 a	1.23 ± 0.06 a
	RHA 50	4.21 ± 0.11 a	26.77 ± 0.17 bc	6.21 ± 0.19 b	0.33 ± 0.11 a	1.10 ± 0.20 ab
	RHA 60	3.42 ± 0.18 c	25.89 ± 0.27 c	7.57 ± 0.25 a	0.27 ± 0.13 b	1.01 ± 0.11 b
Melon	CK	6.33 ± 0.22 c	21.01 ± 0.18 bc	3.32 ± 0.30 a	0.60 ± 0.05 bc	1.73 ± 0.29 b
	RHA 20	6.32 ± 0.24 c	23.15 ± 0.34 b	3.31 ± 0.24 a	0.62 ± 0.02 bc	1.89 ± 0.31 b
	RHA 30	6.57 ± 0.17 b	22.43 ± 0.21 b	3.42 ± 0.17 a	0.64 ± 0.12 b	2.01 ± 0.35 a
	RHA 40	7.61 ± 0.25 a	25.45 ± 0.27 a	3.34 ± 0.20 a	0.73 ± 0.17 a	2.04 ± 0.23 a
	RHA 50	7.58 ± 0.19 a	21.52 ± 0.3 6b	3.32 ± 0.14 a	0.70 ± 0.08 a	1.84 ± 0.19 ab
	RHA 60	6.23 ± 0.20 d	20.30 ± 0.28 c	3.26 ± 0.21 a	0.58 ± 0.10 c	1.80 ± 0.26 b

The mean value ± standard error in the same column with dissimilar letters are significantly different at *P* < 0.05.

In conclusion, adding a proper amount of RHA to the substrate could increase the fruit yield of cucumber and melon, and RHA 40 had the best effect.

## Discussion

Evaluating the nutritional value of RHA holds promise as an initiative to create an appealing alternative for the rice processing industry. This endeavor could offer rice mills a new revenue stream while concurrently addressing the challenge of agricultural waste reduction or elimination. RHA, as a potential supplementary fertilizer source, proves convenient for paddy cultivation. The judicious incorporation of RHA into cultivation substrates has demonstrated its effectiveness in enhancing the physical and chemical properties, significantly promoting plant growth, improving the photosynthetic capacity of plant leaves, and enhancing the quality of plant fruits^23^. Utilizing RHA as a fertilizer not only enhances agricultural productivity but also mitigates the challenge of its disposal^10^.

Soilless cultivation offers several advantages, including reduced susceptibility to pests and diseases, water and fertilizer conservation, freedom from regional constraints, and greater control compared to traditional soil cultivation^24^. Substrate is the main nutrient medium for the growth of soilless plants. Ali et al.^25^ demonstrated that different substrate proportions exert varying effects on plant growth due to differences in water retention, air permeability, and nutrient content. Before cultivation, the water-holding pores, aeration porosity, and total pores of RHA 40 were the largest, which was beneficial to the respiration of plant roots. After cultivation, the bulk density of the cucumber substrate was 0.18–0.25 g cm^−3^, while the aeration porosity was 17.39–26.36%. The bulk density of melon substrate was 0.19–0.27 g cm^−3^, and the aeration porosity was 15.12–21.70%, which was in line with the quality standards of substrate proposed by Ravia^26^ and Wang^27^.The EC value can reflect the soluble salt content in the substrate. If the EC value is too low, the mineral nutrition of the substrate is insufficient; if the EC value is too high, it can cause salt poisoning or salt stress, and crops cannot grow normally^28^. Li et al.^29^ highlighted that soil acidity and alkalinity are fundamental soil characteristics and crucial factors influencing soil fertility and crop growth indicators. Typically, crops thrive in environments with a soil pH ranging from 6.0 to 7.0^30^. The EC values of the substrates of cucumber and melon after cultivation were between 0.32 and 1.72 mS cm^−1^, which was within the range of safe EC values for crop growth (≤ 2.6 mS cm^−1^)^31^, and the pH value of cucumber and melon substrate was between 6.10 and 6.81, which is in line with the pH value range of crop cultivation. However, both the EC and pH values of the cucumber and melon substrates were slightly lower than those before cultivation. This decline may be attributed to the leaching effect of mineral nutrients and water absorbed by the plants during the growth process. The reduction in microbial populations in the medium might be linked to the high-temperature carbonization process of RHA, which tends to eliminate various bacteria. Moreover, the weak alkalinity of RHA creates a favorable, mildly alkaline environment for plant growth. Nevertheless, further studies are warranted to validate these observations.

The root activity reflects the ability of plants to absorb water and nutrients, root volume serves as an indicator of plant robustness, while both dry and fresh weight measurements reflect the accumulation of plant matter^32^. The root-shoot ratio, on the other hand, provides insights into the dry and wet conditions of the root environment and the rate of water utilization^33,34^. Qian et al.^35^ demonstrated that a strong seedling index is indicative of the dry matter accumulation, plant height, stem diameter, and dry and fresh weight of plants. These factors collectively contribute to the strength of the seedling index. In this study, RHA 40 showed the highest dry weight per plant, root volume, root activity, seedling index, plant height, and stem diameter compared with other treatments. Furthermore, RHA 40 significantly enhanced the growth indices of cucumber and melon, thereby fostering the overall growth of these plants.

Photosynthesis plays a crucial role in synthesizing organic substances essential for plant growth, serving as the foundation for plant development^36^. The intensity of photosynthesis is intricately linked to chlorophyll content, and the level of chlorophyll in leaves directly impacts a plant's ability to absorb and convert photosynthesis^17^. There is a certain linear relationship between photosynthetic rate and stomatal conductance, that is, photosynthetic rate increases and stomatal conductance increases, while when photosynthesis is blocked, stomatal conductance decreases, leading to the reduction of CO_2_ entering the leaves^37^. In this study, the photosynthetic pigment content of cucumber and melon reached its maximum under RHA 40. Additionally, *P*n, *G*s, *T*r, and *C*i attained their peaks under RHA 40. These findings suggest that the light energy conversion capability and photosynthetic activity of plant leaves were at their strongest when RHA was added at a 40% ratio.

Seeds can produce rich soluble sugars and proteins during germination, and soluble sugars can provide rich energy for plant growth and development^38^. Sucrose, as a photosynthetic product, is an important link of carbohydrate metabolism in source and sink, and can be mutually transformed with starch^39^. High nitrate content tends to cause physiological adverse effects on plants, thus affecting the growth of fruits and vegetables. In this study, the content of starch and nitrate in fruits of cucumber and melon treated with RHA 40 was the lowest, while the sucrose content, soluble total sugar content, Vc content, and soluble protein content were the highest. This indicated that a certain proportion of RHA could effectively promoting photosynthesis and respiration in the growth and enhancing the ability of plant growth and metabolism. Sucrose phosphate synthase (SPS) and acid invertase (AI) can reflect the ability of sucrose accumulation in plants, and they are one of the main enzymes that make sucrose enter the metabolic pathway^40^. Sucrose synthase (SS) can catalyze the synthesis and decomposition of sucrose, and it is a reversible enzyme^41^. Studies have shown that SPS and SS activities are significantly positively correlated with sucrose content. In the present study, the activities of SPS and SS under RHA 40 significantly increased compared with CK. Sucrose content significantly increased, and the AI activity of cucumber and melon was significantly lower than CK. The transverse diameter, longitudinal diameter, and single fruit weight of the fruit treated with RHA 40 were the highest. In conclusion, the sucrose synthase activity (SS) and sucrose phosphate synthase activity (SPS) of cucumber and melon under RHA 40 are the highest. This outcome is advantageous for the accumulation of carbohydrates, such as sucrose, in the fruit, ultimately enhancing both the yield and quality of the fruit.

## Conclusion

The substrate for soilless cultivation primarily comprises peat. However, given peat's non-renewable nature and associated high costs, the imperative to develop alternative substrates that can partially substitute for peat is paramount. Emerging options, such as rice husk ash (RHA), characterized by wide availability and cost-effectiveness, assume a significant role in addressing this need. This research explored the physical and chemical properties of the composite substrate comprising RHA, analyzing their impact on plant growth, photosynthesis, fruit quality, and carbohydrate metabolism in cucumber and melon plants. The findings indicated that RHA 40 (RHA: peat: vermiculite: perlite = 4:4:1:1 volume ratio) improved substrate ventilation and had a positive impact on the stem diameter, root activity, chlorophyll content, photosynthetic properties and individual fruit weight of cucumber and melon plants. Furthermore, RHA 40 exhibited a notable increase in the levels of sucrose, total soluble sugars, vitamin C, and soluble proteins in the fruits of both cucumber and melon. Moreover, it enhanced the activity of sucrose synthase (SS) and sucrose phosphate synthase (SPS), thereby contributing to the synthesis of sucrose. In summary, RHA 40 demonstrated superior performance as a local growing media for cucumber and melon plant growth, attributed to its outstanding physiochemical characteristics. Additionally, the partial replacement of peat by RHA not only reduces the production cost of soilless culture but also yields positive economic and ecological benefits. For future research on RHA substrate mixed with peat, it is crucial to conduct additional studies aimed at determining the stability of this medium over multiple cultivation cycles. This approach will contribute significantly to building greater confidence in the advantages associated with such growth media.

## Data Availability

All data supporting the findings of this study are available within the paper and its Supplementary Information.
